# Participatory Research with Persons who Experience Mental Illness in Occupational Therapy: A Scoping Review

**DOI:** 10.1177/00084174231212760

**Published:** 2024-01-19

**Authors:** Elham Javadizadeh, Abram Oudshoorn, Lori Letts, Skye Barbic, Chelsea Shanoff, Carrie Anne Marshall

**Keywords:** Mental health, Mental health services, Community-based participatory research, Participatory action research, Occupational therapy, Ergothérapie, recherche participative en milieu communautaire, recherche-action participative, santé mentale, services de santé mentale

## Abstract

**Background.** Persons who experience mental illness also face stigma and discrimination that frequently lead to a loss of ability to exercise autonomy and agency in their lives. **Purpose.** The range and breadth of literature exploring participatory research with persons living with mental illness are unknown in occupational therapy and occupation science. We initiated this study to fill this gap in the existing occupational therapy and occupational science literature. **Method.** Using the method of Arksey and O’Malley, we have conducted a scoping review to identify the range and breadth of literature. A qualitative content analysis was performed. **Findings.** A total of 34 articles were included in the narrative synthesis. The content analysis led to three related themes from the included studies: (1) *coming together*; (2) *unique potential of participatory research*; and (3) *challenges in conducting participatory research*. **Conclusions.** This review highlights that participatory research is well suited to research conducted with persons living with mental illness to support meaningful engagement and minimize stigma throughout the research process. This review can guide future participatory research and practice in occupational therapy and occupational science with persons living with mental illness.

## Résumé

**Description**. Les personnes ayant des maladies mentales font également face à la stigmatisation et à la discrimination, ce qui entraîne souvent une perte de leur capacité à exercer leur autonomie et leur agence dans leur vie. **But.** L'étendue et la portée des recherches explorant la recherche participative avec les personnes vivant avec des maladies mentales restent inconnues dans le domaine de l'ergothérapie et des sciences de l'occupation. Nous avons lancé cette étude afin de combler cette lacune dans la littérature existante en ergothérapie et en sciences de l'occupation. **Méthode**. En utilisant la méthode d'Arksey et O'Malley (2005), nous avons réalisé un étude de porté pour identifier l'étendue et la portée de la littérature. Une analyse de contenu qualitative a été réalisée. **Résultats**. Un total de 34 articles ont été inclus dans la synthèse narrative. L'analyse de contenu a révélé trois thèmes liés issus des études incluses: (1) Se rassembler; (2) Le potentiel unique de la recherche participative; et (3) Les défis de la réalisation de la recherche participative. **Conclusions**. Cette revue met en évidence que la recherche participative est particulièrement adaptée à la recherche menée auprès des personnes vivant avec des maladies mentales, afin de favoriser leur engagement significatif et de réduire la stigmatisation tout au long du processus de recherche. Cette revue peut servir de guide pour orienter les futures recherches participatives et les pratiques en ergothérapie et en sciences de l'occupation avec les personnes vivant avec des maladies mentales.

## Introduction

Participatory research is a broad concept encompassing a wide variety of approaches to empower community members to participate in research and thereby engage them in making decisions that influence their lives (Jason et al., 2004). According to Balcazar et al. (2006), a participatory approach includes recognition of persons with lived and living experiences as research collaborators and engages them as active participants in all research phases, including defining the problem to be addressed, data collection, interpretation, and dissemination of findings. One of the significant differences between participatory approaches and other research models is that community members are involved in shaping research questions, and the traditional roles reserved for researchers, consumers, and service providers are reconceptualized (Balcazar et al., 1998).

Participatory research is an umbrella term for a range of approaches that incorporate individuals with lived experiences, service providers, and stakeholders throughout the research process (Cargo & Mercer, 2008). Green et al. (1995, p. 5) defined participatory research as “systematic inquiry, with the collaboration of those affected by the issue being studied, for purposes of education and taking action or effecting change.” While participatory action research is the most familiar term to most researchers (MacDonald, 2012), additional parallel approaches include community-based participatory research (CBPR), action research, and experience-based co-design (EBCD), among others. The purpose of action research is to improve the capacity and practice of the researcher instead of producing theoretical knowledge and does not always engage research participants in the study (Elliot, 1991). In participatory research and CBPR, researchers and community co-researchers collaborate to change a social reality (Whyte, 1998). Participatory action research is the combination of action research and participatory research with the goal of improving the capacity and practice of researchers and changing social reality through participation (Elliot, 1991).

Letts (2003) indicates that two key concepts of occupational therapy have a strong link with participatory research principles: client-centred practice and occupation. Participatory research, like client-centred occupational therapy, emphasizes bringing participants to the process of research and using their expertise to develop knowledge. In both participatory research and client-centred occupational therapy, the knowledge that participants bring to the encounter is crucial (Letts, 2003). The core values of client-centredness, such as the right of a person to choose occupations, are congruent with the holistic view of occupational therapy and the theoretical framework of occupational performance (Law et al., 1995).

A second connection between participatory research and occupational therapy is the concept of occupation (Letts, 2003). Occupation has a significant role in promoting health (Egan, 2022). Participatory research can be thought of as an occupation in itself in that the concept of “action” is considered a key component of the research process (Letts, 2003). In occupational therapy and occupational science literature, participatory approaches have been conducted with persons with different health conditions, including persons living with physical disabilities (Bhagwanjee & Stewart, 1999), adults with mental health conditions (Andonian, 2010), older adults (Andonian & MacRae, 2011), occupational therapists (Egan et al., 2004), children with mental health disorder (Greco et al., 2017), immigrants and refugees (Suarez-Balcazar et al., 2018), and low-income communities and individuals with chronic health conditions (Wang, 1999).

### The Relevance of Participatory Research for Persons Living with Mental Illness

The unique strengths and challenges faced by diverse persons who experience mental illness present an important opportunity for using participatory approaches that take an occupational perspective. Mental illness refers to a wide range of conditions that affect cognition, emotion, and behaviour that can create challenges for occupational participation (Manderscheid et al., 2010). The importance of involving diverse individuals with mental illness in the development of approaches that are co-designed has been broadly recognized as an ideal approach in refining and creating innovative health systems (Chodo, 2015). According to Bassman (2001), persons living with mental illness have historically lacked a voice, and they are infrequently involved in decision-making about the mental health services. The historical oppression faced by persons living with mental illness makes this population a key group with which to conduct participatory research, as listening to and acting upon their perspectives is an opportunity to reconcile past oppression (Kleintjes, 2012).

### The Current Study

Participatory approaches have been used in a variety of ways within occupational therapy and occupational science; however, little is known about the scope of participatory research conducted with persons living with mental illness specifically. Understanding the breadth of literature in this area will provide a foundation on which to advance research and knowledge in action. Although there are number of reviews on participatory research and health in other disciplines (Stacciarini et al., 2011) or with other populations (Rustage et al., 2021), to our knowledge, there are no existing systematic or scoping reviews which have been conducted to synthesize literature on participatory research with persons living with mental illness in occupational therapy and occupation science. To fill this gap in the existing literature, we conducted a scoping review guided by the research question: *What is the scope of participatory research within the field of occupational therapy and occupational science regarding persons who experience mental illness?*

## Method

We conducted a scoping review following the methodological framework outlined by Arksey and O’Malley (2005) using the PRISMA-ScR guidelines (Tricco et al., 2018). Arksey and O’Malley's framework encompasses five steps, which are described below.

### Search Strategy

We initially deployed our search in November 2021 and updated our search in December 2022. Our search combined the concepts of participatory research (*e.g.*, participatory action research, photovoice) and occupational therapy and occupational science (occupational therapy*, occupational science) using a Boolean “AND.” The search strategy was deployed in nine databases (Medline, Embase, CINAHL, Sociological Abstracts, Nursing and Allied Health, Social Service Abstracts, Social Work Abstracts, Cochrane and PychoINFO). A sample of our search, deployed in Medline, is provided in Appendix 1.

#### Inclusion and Exclusion Criteria

We included articles that: (a) used participatory research approaches; (b) pertained to persons who experience mental illness or mental health challenges; (c) were published in English or Persian; (d) published in all years; (e) pertaining to persons of all ages; (f) scoping and systematic reviews of empirical studies; and (g) had been published within the field of occupational therapy or occupational science (*e.g.*, it was published in an occupational science or occupational therapy journal or was written by a first or last author identifying as an occupational therapist or occupational scientist).

We excluded articles under the following criteria: (a) action research studies that were not explicitly participatory; (b) conference abstracts; and (c) dissertations and theses.

#### Study Selection

We uploaded our searches from each database into Covidence™ (*Covidence systematic review software, 2022*), a software program that allows for collaborative review and data extraction. Two independent raters (EJ and CS) subsequently screened titles and abstracts by comparing each against the inclusion and exclusion criteria identified above. The full texts of studies included at the title and abstract screening stage were uploaded, and two independent raters read each paper in full, comparing each against the set of inclusion and exclusion criteria to determine eligibility. Conflicts arising during either of these stages were resolved by consensus. When the conflict could not be resolved using two independent raters, we sought the input of a third rater (CM).

#### Data Extraction

Using a custom data extraction form developed in Covidence (*Covidence systematic review software, 2022*), we extracted the following information from each included study: author(s); year of publication; study design; methodology; participant type (*e.g.*, persons with mental illness, occupational therapists, parents, *etc*.); clinical characteristics of participants; number of participants; demographic characteristics of participants; country of authors; and level of participation. To determine the level of participation of persons with lived experiences of mental illness in each study, we used a framework introduced by Kindon et al. (2007). This framework identifies key actions taken by researchers to involve persons with lived experience in a participatory study, and thus, provides an opportunity to measure the level of participation used in participatory studies. Further, Kindon et al. (2007) introduced five recurrent stages of action and reflection, including establishing relationships and a common agenda with all stakeholders; collectively designing research processes and tools; collaboratively analyzing the findings; working on feeding research back to all participants; and collectively identifying future research and impacts. We used the information provided by the authors of the included studies in the methodology section of each paper to identify whether they reported the process of action and reflection.

#### Narrative Synthesis

Qualitative content analysis (Graneheim & Lundman, 2004) was employed to code statements in the included studies pertaining to the research question using Dedoose (*Dedoose*, 202*1*), a qualitative data management program. This analysis involved reading through the full texts of the included articles to identify statements that were related to the research question. We then coded each statement inductively, followed by creating categories and sub-categories to generate themes and sub-themes that were related to the research question.

### Findings

**Figure 1.**
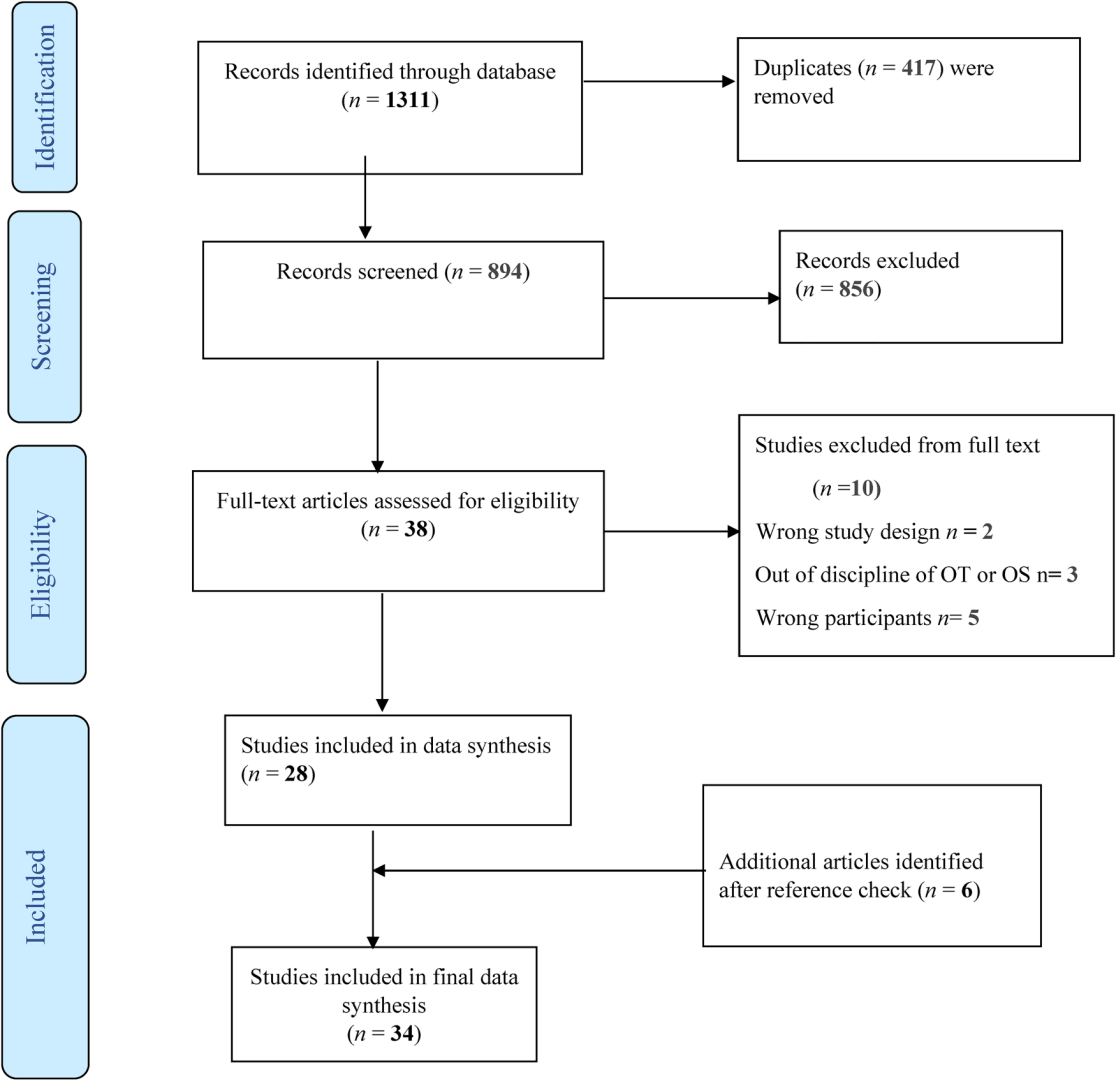
PRISMA diagram.

The search yielded 1,311 citations. A total of 894 studies remained after removing 417 duplicates. 856 studies were eliminated during the title and abstract screening. We calculated Kappa statistics to assess the agreement among the raters for both the title and abstract screening and full-text review phases. Inter-rater reliability for the title abstract screening stage was 0.47, which demonstrates a “moderate” strength of agreement according to Ashby (1991). Thirty-eight articles were subjected to full-text review. For the full-text review phase, inter-rater reliability was 0.55, also demonstrating a “moderate” strength of agreement according to Ashby (1991). A total of 28 articles met the criteria for inclusion, and six studies were added from the reference lists of the included articles, which resulted in 34 studies in the final review. See Figure 1 for the summary of the study selection process and reasons for exclusion.

Of the 34 articles included in this review, 23 (76.4%) used qualitative methods, two (5.8%) studies used mixed methods, five (14.7%) were expert opinion papers, and one was a scoping review. Nearly half of the included studies (52.9%) were conducted in Canada and United Kingdom (nine studies each country), followed by the United States (*n* = 8; 23.5%), Australia (*n* = 5; 14.7%), Singapore (*n* = 1; 2.9%), and South Africa (*n* = 1; 2.9%), and internationally (North America, South America, Europe, and Africa) (*n* = 1; 2.9%). Studies included in this review spanned from 1993 to 2022. A total of 22 (73.5%) studies were conducted after 2010. Twenty-two articles (64.7%) were published in occupational therapy journals, 11 (32.3%) in interdisciplinary journals, and one (2.9%) in an occupational science journal. The characteristics of individual studies included in this review are provided in Table 1.

**Table 1:** Description of the Included Studies (n = 34)

Authors	Study design	Methodology^a^	Methods	Types of participants^a^	Clinical characteristics of the sample	Number of participants	Demographic characteristics of the sample	Journal discipline	Country
Andonian (2010)	Qualitative	Participatory research	Photovoice	People with mental health issues	Bipolar affective disorder (*n* = 2)	5	**Age**: 21–56, **Gender**: *n* = 3 men; *n* = 2 women. **Race/ethnicity**: Caucasian (*n* = 3); Japanese and Filipina (*n* = 1); African American and Native American (*n* = 1). **LGBTQ2**: Unspecified	Occupational therapy	United States
Arblaster et al. (2018)	Qualitative	Participatory	Delphi method	Mental health consumers	Unspecified	14	**Age**: 20–59 **Gender**: *n* = 3 men; *n* = 11 women **Race/ethnicity**: Aboriginal (*n* = 1) **LGBTQ2**: Unspecified	Occupational therapy	Australia
Arblaster et al. (2019)	Qualitative	Participatory research approach	Interview	Mental health consumers	Unspecified	16	**Age**: 27–67 **Gender**: *n* = 7 men; *n *= 9 women **Race/ethnicity**: Aboriginal (*n* = 1); non-Aboriginal (*n* = 15) **LGBTQ2**: Unspecified	Occupational therapy	Australia
Baker and Procter (2014)	Qualitative	Participatory action research	Focus group	Occupational therapy and adults affected by mental illness	Major depression (*n* = 9); bipolar disorder (*n *= 7); anxiety disorder (*n* = 3); schizoaffective disorder (*n* = 2); borderline personality disorder (*n* = 2); schizophrenia (*n* = 2); obsessive compulsive disorder (*n* = 1)	16	**Age**: 39-59 **Gende**r: *n* = 5 men; *n* = 11 women **Race/ethnicity**: Unspecified **LGBTQ2**: Unspecified	Occupational therapy	Australia
Briken and Bryant (2019)	Reflection paper	Participatory research	Photovoice	Occupational therapy staff and service users from the acute mental health unit	Unspecified	Unspecified	Unspecified	Occupational therapy	United Kingdom
Bryant et al. (2010)	Qualitative	Participatory action research	Focus group	Mental health service users and staff	Unspecified	At least ten people attended every meeting	Unspecified	Interdisciplinary	United Kingdom
Bryant et al. (2012)	Qualitative	Participatory action research	Researching Psychosis together group	People using local mental health services, an assistant, and an occupational therapist from local services, and an academician	Unspecified	8	**Age**: Unspecified **Gender**: *n* = 3 men; *n* = 5 women **Race/ethnicity**: Unspecified **LGBTQ2**: Unspecified	Interdisciplinary	United Kingdom
Bryant et al. (2019)	Qualitative	Participatory action research		Mental health service users, occupational therapy students, health professionals, and academicians	Unspecified	Unspecified	Unspecified	Interdisciplinary	United Kingdom
Bryce (2012)	Qualitative	Auto ethnography	Photovoice	People receiving services from a specialist outreach mental health team	Unspecified	Unspecified	Unspecified	Interdisciplinary	United Kingdom
Clark et al. (1993)	NA	Expert opinion		NA	NA	NA	NA	Occupational therapy	Canada
Anderson Clarke and Warner (2016)	Qualitative	Phenomenological research	Photovoice	Individuals diagnosed with mental illness	Depression (*n* = 1); schizophrenia (*n* = 1); bipolar (*n* = 1); depression & anxiety (*n *= 1); depression, schizophrenia, anxiety, substance abuse (*n* = 1); personality disorder, depression, anxiety, intellectual disability (*n* = 1); bipolar, anxiety, dissociative identity disorder (*n* = 1); personality disorder, bipolar, depression, schizophrenia, anxiety (*n* = 1)	8	**Age**: 27-63 **Gender**: *n* = 4 men; *n* = 4 women **Race/ethnicity**: White (*n* = 5); Black (*n* = 1); White, Hispanic, and multiracial (*n* = 1); Other *(n* = 1) **LGBTQ2**: Unspecified	Occupational therapy	United States
Cockburn and Trentham (2002)	NA	Expert opinion	-	NA	NA	NA	NA	Occupational therapy	Canada
Dixon et al. (2022)	Qualitative	Participatory research	Photovoice	Women with mental health issues	Unspecified	12	**Age**: Unspecified **Gender**: All women **Race/ethnicity**: Unspecified **LGBTQ2**: Unspecified	Interdisciplinary	Australia
Doll and Brady (2013)	Mixed Methods	Community-based participatory research	Focus group	Students recruited by teachers as potential risk for suicide	Unspecified	Unspecified	**Age**: Unspecified **Gender**: Unspecified **Race/ethnicity**: All participants were Native Americans **LGBTQ2**: Unspecified	Occupational therapy	United States
Greco et al. (2017)	Qualitative	Narrative-phenomenology	Photovoice	Children in a school-based psychiatric setting	Attention deficit disorder, oppositional defiant disorder, conduct disorder, anxiety disorder, and/or learning delays	4	**Age**: 9–10 **Gender**: *n* = 2 men; *n* = 2 women **Race/ethnicity**: Unspecified **LGBTQ2**: Unspecified	Occupational therapy	Canada
Ingolia and Barrett (2019)	Qualitative	Participatory research	Photovoice	Mothers of children with complex trauma	Unspecified	6	**Age**: The mothers’ ages ranged from young adulthood to middle age **Gender**: *n* = 6 women **Race/ethnicity**: (*n *= 5) **Caucasian**: (*n* = 1) Latina **LGBTQ2**: Unspecified	Occupational therapy	United States
Letts (2003)	NA	Expert opinion	-	NA	NA	NA	NA	Occupational therapy	Canada
Makdisi et al. (2013)	Qualitative	Participatory research	Focus group	People with experience of psychosis, facilitated by service user researchers and an MSc student	Unspecified	13	**Age**: Unspecified **Gender**: *n* = 8 men; *n* = 5 women **Race/ethnicity**: Unspecified **LGBTQ2**: Unspecified	Occupational therapy	United Kingdom
Maniam et al. (2016)	Qualitative	Participatory research	Photovoice	People who have been diagnosed with first-episode or at risk of psychosis	Schizophrenia (*n* = 5); bipolar disorder (*n* = 2); delusional disorder with OCD (*n* = 1); psychosis not otherwise specified (*n* = 1); at risk (*n* = 2)	11	**Age:** 21-38 **Gender**: *n* = 4 men; *n* = 7 women **Race/ethnicity**: Unspecified **LGBTQ2**: Unspecified	Occupational therapy	Singapore
Mirza et al. (2008)	Qualitative	Participatory action research	Focus group	People with psychiatric disabilities	Unspecified	8	**Age**: All the participants were middle-aged **Gender**: *n* = 6 men; *n* = 2 women **Race/ethnicity**: 7 African Americans (*n* = 7), 1 Caucasian (*n* = 1)/Black (*n* = 7); White (*n* = 1) **LGBTQ2**: Unspecified	Interdisciplinary	United States
O’Brien et al. (2021)		Scoping review	Arksey and O'Malley's framework	NA	NA	NA	NA	Interdisciplinary	Australia
Pettican et al. (2021)	Qualitative	Participatory action research	World Café events	Persons who experience mental distress	Unspecified	23	**Age**: Unspecified **Gender**: *n* = 21 men; *n* = 2 women **Race/ethnicity**: Unspecified **LGBTQ2**: Unspecified	Occupational science	United Kingdom
Pettican et al. (2022)	Qualitative	Participatory action research	World Café events	Persons who experience mental distress	Unspecified	8	Unspecified	Occupational therapy	United Kingdom
Rempfer and Knott (2002)	NA	Expert opinion		NA	NA	NA	NA	Occupational therapy	United States
**Authors**	**Study design**	**Methodology**	**Methods**	**Types of participants^a^**	**Clinical characteristics of the sample**	**Number of participants**	**Demographic characteristics of the sample**	**Journal discipline**	**Country**
Russinova et al. (2018)	Qualitative	Participatory research	Photovoice	Persons with mental health challenges	Schizophrenia spectrum disorder (*n* = 13)	43	**Age**: <40 (28%), 40–55 (35%), <55 (37%) **Gender**: *n* = 13 men; *n* = 30 women **Race/ethnicity**: White (*n* = 31), not White (*n* = 12) **LGBTQ2**: Unspecified	Interdisciplinary	United States
Schwartz et al. (2013)	Qualitative	Participatory action research and narrative phenomenological methodology	Focus group	Five consumers of mental health services, three occupational therapists, one psychiatrist, and a clinician-researcher affiliated with a university	Unspecified	10	Unspecified	Interdisciplinary	Canada
Schwartz et al. (2020)	Qualitative	Participatory research	Stakeholder-engaged approach	Mental health clinicians (*n* = 10), peer providers (*n* = 9), and transition specialists (*n* = 20)	3 had diagnoses of autism spectrum disorder and anxiety and/or depression (*n *= 3); diverse intellectual/developmental disabilities and mental health conditions (*n *= 3)	39	**Age**: Unspecified **Gender**: *n* = 6 men; *n* = 33 women **Race/ethnicity**: white (*n* = 33); African American (*n* = 3); Asian (*n* = 1); other (*n* = 2) **LGBTQ2**: No one had non-binary/other gender identities	Interdisciplinary	United States
Shaffer et al. (2020)	Mixed methods	Pre-test, post-test pilot design, and a participatory action approach	Interview	Parents of special needs children	Unspecified	11	**Age**: 20–59 **Gender**: *n* = 11 women **Race/ethnicity**: Unspecified **LGBTQ2**: Unspecified	Interdisciplinary	North America (5), South America (2), Europe (1), Africa (3)
Smith and Suto (2014)	Qualitative	Community-based participatory research	Interview, Focus group	Patients in acute psychiatry units	Unspecified	7	**Age**: Early 20 s to Late 60s **Gende**r: *n* = 5 men; *n* = 2 women **Race/ethnicity**: Unspecified **LGBTQ2:** Unspecified	Occupational therapy	Canada
Suto et al. (2021)	Qualitative	Community-based participatory research	Focus group, interview, aping activities	Individuals living with serious mental illness and/or addictions	ADHD, anxiety disorder, bipolar disorder, depression, schizoaffective disorder, schizophrenia, substance misuse	23	**Age**: 32–67 **Gender**: *n* = 15 men; *n* = 8 women **Race/ethnicity**: Unspecified **LGBTQ2**: Unspecified	Occupational therapy	Canada
Suto and Smith (2014)	Qualitative	Community-based participatory research	Interview, focus group	Acute-based mental health professionals	Unspecified	8	Unspecified	Occupational therapy	Canada
Townsend et al. (2000)	Qualitative	Ethnography + Participatory research	Interview	People with long-term mental illness	Unspecified	Average attendance has ranged from 8 to 12 participants per meeting	Unspecified	Occupational therapy	Canada
Tsatsi and Plastow (2021)	Qualitative	Participatory action research	Interview	Mental health care users	Unspecified	9	**Age**: Unspecified **Gender**: All men **Race/ethnicity**: All black Africans **LGBTQ2**: Unspecified	Occupational therapy	South Africa
Wimpenny et al. (2010)	Qualitative	Participatory action research	Focus group	Occupational therapists	Unspecified	15	Unspecified	Occupational therapy	United Kingdom

^a^ Terminologies/descriptions are based on the information provided in the specific papers.

**Figure 2.**
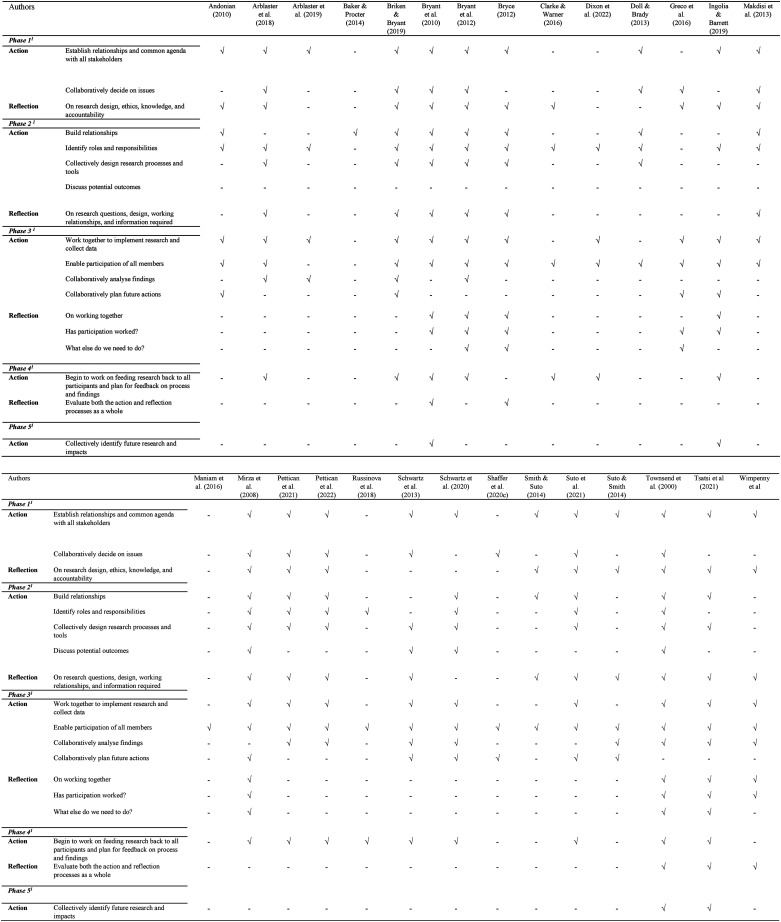
Level of participation of the included studies (*N* = 28).

The level of participation in the included empirical studies (*n* = 28) according to the framework provided by Kindon (33) is provided in Figure 2. In 14 of the included studies (41.1%), the authors and participants collaboratively decided on the focus of the study. Fourteen studies (41.1%) collectively designed research processes and tools. In 12 of the included studies (35.2%), participants and researchers collaboratively analyzed the findings. Only four of the included studies (11.7%) collectively identified future research and impacts.

### Narrative Synthesis

We generated three themes in our content analysis: (a) coming together; (b) unique potentials of participatory research; and (c) challenges of conducting participatory research. Articles associated with these themes and associated sub-themes are presented in Table 2.

**Table 2:** Themes and Sub-Themes Represented by the Included Studies

Theme	Sub-themes	Studies included
Coming together (*n *= 23)		Arblaster et al., 2018; Arblaster et al., 2019; Baker & Procter, 2014; Birken & Bryant, 2019; Bryant et al., 2010; Bryant et al., 2012; Bryant et al., 2019; Bryce, 2012; Anderson Clarke & Warner, 2016; Doll & Brady, 2013; Ingolia & Barrett, 2019; Makdisi et al., 2013; Maniam et al., 2016; Mirza et al., 2008; O’Brien et al., 2021; Rempfer & Knott, 2002; Schwartz et al., 2013; Schwartz et al., 2020; Shaffer et al., 2020; Smith & Suto, 2014; Suto & Smith, 2014; Suto et al., 2021; Tsatsi & Plastow, 2021
	Achieving a shared understanding (*n *= 19)	Arblaster et al., 2018; Arblaster et al., 2019; Baker & Procter, 2014; Birken & Bryant, 2019; Bryant et al., 2012; Bryant et al., 2019; Bryce, 2012; Anderson Clarke & Warner, 2016; Doll & Brady, 2013; Ingolia & Barrett, 2019; Makdisi et al., 2013; Maniam et al., 2016; Mirza et al., 2008; O’Brien et al., 2021; Rempfer & Knott, 2002; Schwartz et al., 2013; Schwartz et al., 2020; Shaffer et al., 2020; Suto et al., 2021
	Effective communication (*n *= 10)	Arblaster et al., 2019; Bryant et al., 2010; Bryant et al., 2012; Bryant et al., 2019; O’Brien et al., 2021; Schwartz et al., 2013; Schwartz et al., 2020; Smith & Suto, 2014; Suto & Smith, 2014; Tsatsi & Plastow, 2021
Unique potentials of participatory research (*n *= 23)		Andonian, 2010; Arblaster et al., 2019; Bryant et al., 2010; Bryant et al., 2012; Bryant et al., 2019; Bryce, 2012; Anderson Clarke & Warner, 2016; Clark et al.,1993; Cockburn & Trentham, 2002; Dixon et al., 2022; Doll & Brady, 2013; Greco et al., 2017; Ingolia & Barrett, 2019; Letts, 2003; Maniam et al., 2016; O’Brien et al., 2021; Pettican et al., 2021; Pettican et al., 2022; Rempfer & Knott, 2002; Russinova et al., 2018; Townsend et al., 2000; Tsatsi & Plastow, 2021; Suto et al., 2021
	Empowerment (*n *= 13)	Andonian, 2010; Bryant et al., 2010; Bryant et al., 2012; Bryant et al., 2019; Anderson Clarke & Warner, 2016; Cockburn & Trentham, 2002; Greco et al., 2017; Ingolia & Barrett, 2019; Maniam et al., 2016; Rempfer & Knott, 2002; Suto et al., 2021; Townsend et al., 2000; Tsatsi & Plastow, 2021
	Sharing power (*n *= 10)	Bryant et al., 2010; Bryant et al., 2019; Bryce, 2012; Clark et al., 1993; Cockburn & Trentham, 2002; Greco et al., 2017; Letts, 2003; O’Brien et al., 2021; Rempfer & Knott, 2002; Townsend et al., 2000
	Stigma resistance (*n *= 4)	Bryant et al., 2019; Rempfer & Knott, 2002; Russinova et al., 2018; Townsend et al., 2000
	Expressing what matters (*n *= 14)	Arblaster et al., 2019; Andonian, 2010; Bryant et al., 2010; Bryce, 2012; Clark et al.,1993; Dixon et al., 2022; Doll & Brady, 2013; Greco et al., 2017; Maniam et al., 2016; Pettican et al., 2021; Pettican et al., 2022; Rempfer & Knott, 2002; Townsend et al., 2000; Tsatsi & Plastow, 2021
Challenges of conducting participatory research (*n *= 11)		Andonian, 2010; Bryant et al., 2010; Bryant et al., 2012; Bryant et al., 2019; Cockburn & Trentham, 2002; Letts, 2003; Maniam et al., 2016; Rempfer & Knott, 2002; Townsend et al., 2000; Tsatsi & Plastow, 2021; Wimpenny et al., 2010

#### “Coming Together”

The concept of “coming together” was explored in 23 articles (67.64%) in this review (see Table 2). Broadly, coming together can be defined as a context that brings experts by experience (mental health service users), experts by profession (service providers), and researchers who work together and share power to improve the experience of mental health service users. These articles noted that “coming together” is possible through (a) achieving a shared understanding and (b) effective communication.

##### Achieving a Shared Understanding

The value of achieving a shared understanding between persons with mental illness and service providers and researchers was discussed in 19 of the included studies in this review (See Table 2). Bryant (Bryant et al., 2012) believed that in the context of participatory research, both occupational therapists and persons with mental illnesses have something to learn, and involving all stakeholders in research creates space for all to learn from each other and achieve a shared sense of purpose. In Maniam's study (2016), photovoice as a participatory method was identified as a unique tool for occupational therapists to hold dialogs with their clients. The findings of eight studies found that there is a mismatch between the priorities and goals of people accessing mental health services and the focus of occupational therapy mental health services themselves (Anderson Clarke & Warner, 2016; Arblaster et al., 2018; Birken & Bryant, 2019; Ingolia & Barrett, 2019; Mirza et al., 2008; Rempfer & Knott, 2002; Schwartz et al., 2013; Shaffer et al., 2020). Schwartz et al. (2013) pointed out that the conflict between values of beneficence in medical ethics increases the tension between the occupational therapist and consumer values. In Arblaster's study (2018), it was discussed that occupational therapists usually value clinical reasoning and evidence-based interventions, while mental health consumers want therapists to understand and value their perspective and support their choice. Arblaster et al. (2018) found that “consumer’s emphasis on human connection and ‘doing with’, and a therapist’s emphasis on ‘doing to’.” The participants in Arblaster's study (2018) believed that human connection is a core value that underpinned the recovery-oriented occupational therapy practice. Several studies (*n* = 11) clearly expressed the need for service providers such as occupational therapists to better understand the needs of persons with mental illness and validate their stories in practice and research (Arblaster et al., 2018; Baker & Procter, 2014; Bryant et al., 2012; Bryant et al., 2019; Bryce, 2012; Doll & Brady, 2013; Makdisi et al., 2013; O’Brien et al., 2021; Schwartz et al., 2013; Schwartz et al., 2020; Suto et al., 2021). Collaborative relationship (Arblaster et al., 2019; Bryant et al., 2012; Suto et al., 2021), learning from lived experiences (Arblaster et al., 2018; Baker & Procter, 2014; Makdisi et al., 2013), professional self-reflection, critically examining power inequity (Arblaster et al., 2019), being open to different knowledge (Bryant et al., 2019), and using co-design protocols (O’Brien et al., 2021) were mentioned as strategies to build up a shared understanding of issues related to mental illness in occupational therapy literature.

##### Effective Communication

Considerations for effective communication between mental health consumers and occupational therapists were discussed in ten participatory studies (see Table 2). Participants in four studies (Arblaster et al., 2019; Bryant et al., 2019; Smith & Suto, 2014; Suto & Smith, 2014) suggested that occupational therapists need to engage in self-reflection to find effective ways of building a therapeutic alliance with persons living with mental illness. The value of informal and non-clinical communication style in the context of participatory projects was identified as a priority in three studies (Bryant et al., 2012; Schwartz et al., 2020; Tsatsi & Plastow, 2021). Being a co-researcher in a non-clinical context was important for changing how everyone communicated (Bryant et al., 2012). Some participants felt that communication would be easier if professionals could “just take off their labels and see what's going on” (Schwartz et al., 2013, p. 115). In contrast, some service providers believed that setting clear boundaries and expectations can foster trust between mental health consumers and service providers (Schwartz et al., 2020). Building culturally sensitive relationships with co-researchers before starting the co-design research projects was also highlighted in O’Brien's study (2021).

### “Unique Potentials of Participatory Research”

Twenty-three articles (67.6%) included in this review identified the unique potential of participatory research for individuals who experience mental illness (see Table 2). The findings of these articles were divided into the following sub-themes: (a) empowerment; (b) sharing power; (c) expressing what matters; and (d) stigma resistance.

#### Empowerment

The experience of collaborating in participatory research created a potential feeling of empowerment for occupational therapy service users in 13 studies (see Table 2). Having an opportunity to learn from each other, expand the sense of meaning, become more vocal, share expertise with a greater audience, improve self-efficacy, feel a sense of mastery and achievement, achieve acceptance and hope, and take action towards facilitating change in their environment were factors provided in the context of participatory research that helped participants feel empowered (Andonian, 2010; Bryant et al., 2012; Bryant et al., 2019; Maniam et al., 2016; Rempfer & Knott, 2002; Tsatsi & Plastow, 2021).

#### Sharing Power

Ten of the included studies demonstrated that power could be shared between individuals with mental illness, mental health professionals, and researchers through conducting participatory studies where the traditional roles of “patient” *versus* “professional” are broken down (see Table 2). In Greco's study (2017), children living with mental illness had the opportunity to take control using photovoice methods, which reduced the power imbalance between children, adults, and researchers. Townsend et al. (2000) suggested that occupational therapists and other professionals need to extend the notion of person-centredness in the context of research in order to reduce power inequity.

#### Stigma Resistance

Participatory research was identified as a potential tool to resist stigma for persons with mental illness in four studies (see Table 2). These studies highlighted the potential of participatory research to increase the knowledge of stigma, and advocate against it. In particular, Russinova et al. (2018) suggested that photovoice was a transformative tool to reduce the impact of prejudice and discrimination toward persons with mental illness and can translate the experience of stigma by presenting it both visually and conceptually. Russinova et al. (2018) believed that photovoice allows persons with mental illness to express their experiences of stigma in a way that feels psychologically safe.

#### Expressing What Matters

Fourteen of the included articles pointed out that participatory approaches enable individuals living with mental illness to express what really matters to them to create relevant and meaningful knowledge (see Table 2). Three of the included studies (Pettican et al., 2021; Pettican et al., 2022; Tsatsi & Plastow, 2021) suggested that involving persons with mental illness in conducting research can assist both mental health consumers and mental health professionals such as occupational therapists to identify important ideas and the desired direction for change. In two studies, authors (Dixon et al., 2022; Rempfer & Knott, 2002) noted that including the voice of mental health consumers helps occupational therapists access the knowledge not represented in the scientific literature. Arblaster et al. (2019) suggested using participatory methods within the field of occupational therapy to ensure that focus of occupation is balanced with the lived experience concerns of persons living with mental illness. Greco et al. (2017) believed that the participatory philosophy of the photovoice method can help children with mental health disorders who use occupational therapy services to generate new domains for self-report measures and report what matters to them.

### “Challenges in Conducting Participatory Research”

The challenges of conducting participatory research were documented in 11 (32.3%) of the included studies (see Table 2). One of the concerns noted by two authors (Bryant et al., 2019; Letts, 2003) was the process for obtaining ethical approval for studies in which service user researchers were also the subjects. In Bryant's study (2019), a university was unfamiliar with the idea of service user co-investigators, and there were mutual misunderstandings throughout the process of seeking ethical approval. Townsend (2000) believed that expectations of research policies, ethical and funding guidelines, and research presentations and publications create barriers to the inclusion of persons living with mental illness and minimize experiential knowledge. The idea of involving service users in research was described was difficult to achieve because the majority of participatory research agendas are determined by academicians and/or service providers, not service users (Bryant et al., 2010). Finding common ground (Bryant et al., 2019), finding the focus of research (Townsend et al., 2000), and disagreement about ownership (Bryant et al., 2019) and leadership (Letts, 2003) of the project were described as challenges associated with conducting participatory research. Two of the included studies made the point that participatory research is time-consuming and needs more resources than the traditional research methods (Letts, 2003; Rempfer & Knott, 2002). In one study, maintaining the enthusiasm and involvement of people accessing mental health services in all project stages became a concern (Cockburn & Trentham, 2002).

In four studies, participants also experienced individual challenges with the participatory projects in which they were involved (Bryant et al., 2010; Bryant et al., 2012; Cockburn & Trentham, 2002; Maniam et al., 2016). Fear and anxiety during focus groups (Bryant et al., 2012), being unfamiliar with the occupation of research (Bryant et al., 2010), being an active decision-maker (Cockburn & Trentham, 2002), fear of being judged, technical difficulties such as working with a camera (in the instance of photovoice), and fear of not completing the project (Maniam et al., 2016) were reported. Participants in Tsatsi's study (2021), believed that their involvement in the scientific part of the research process was limited because they were only involved in the outcome formulation, initial data analysis, and taking action. Andonian (2010) discussed that selection bias in participatory approaches is assumed because the participants willing to be involved in research projects and have an active role might be different from those not comfortable speaking in group settings.

## Discussion

This scoping review aimed to identify the range of research exploring participatory approaches with persons living with mental illness within the fields of occupational therapy and occupational science. Our findings suggest that involving persons with lived and living experiences of mental illness in all stages of the research process can provide several benefits, for all people involved in the research, including researchers, health professionals, and mental health consumers, while also introducing some challenges. The role of co-design and providing opportunities for persons with mental illness to take on leadership roles in health system planning, evaluation, and research is critical for contributing to efforts aimed at reconciling the historical oppression faced by this population. This includes creating the opportunity to identify important research questions, share knowledge, and participate in all stages of the research process, including analysis, interpretation of results, and mobilizing findings into practice and policy, as these are common practices in participatory approaches. However, our review also found barriers to conducting this type of research with this population, including time to do the research and work within institutional challenges that prevent such studies taking place in the first place. We encourage future occupational therapy and occupational science research to document these challenges and propose solutions for the field for how best to navigate institutional challenges, such as ethics/human resources and funding.

The findings of participatory research included in this review highlighted the risk of misalignment between mental health consumers’ priorities and service providers’ goals in practice and research. In one of the included studies in this review, Arblaster et al. (2018) found that human connection and “doing with” are emphasized by mental health consumers, while the focus of mental health professionals is “doing to.” This is consistent with the existing literature, which suggests that occupational engagement of individuals is motivated by the need to belong and connect with others (Andonian & MacRae, 2011; Berger, 2011). We believe that this misalignment should be addressed by working alongside persons who experience mental illness to generate a shared understanding of mental illness and effective approaches for service provision. In research, occupational therapists and occupational scientists can address a historical lack of collaboration with service users by bringing together researchers, service providers, and persons with lived experiences to generate relevant occupation-focused research questions. This suggestion builds on recommendations in previous studies, which suggest that mental health consumers and mental health professionals need to work together to achieve a shared understanding of diagnosis, prognosis, and recovery (McCabe et al., 2013; Papageorgiou et al., 2017).

The findings of this review reveal the unique potential of participatory research for persons living with mental illness. In this review, participants felt empowered by engaging in research projects and being active decision-makers. Clark et al. (1993) believed that involving persons in occupational therapy research is supported by values of occupational therapy practice, in which involvement in decision making is crucial. Given the complexities of environments in which participatory research is conducted, this review suggests that occupational therapy researchers should apply a pragmatic approach that aligns with the core assumptions, principles, and values of occupational therapy (Ikiugu & Schultz, 2006). By doing so, they can effectively support the self-determination of individuals living with mental illness and provide opportunities for practicing social justice.

Collective empowerment aims that characterize participatory research are congruent with occupational therapy's aim to enable empowerment through occupation (Townsend et al., 2000).

This study also demonstrates that research can be an important occupation for marginalized populations such as persons living with mental illness. There is an opportunity to conduct participatory research focused specifically on the occupational injustices faced by this population and identifying opportunities for mitigating these injustices in collaboration with persons with lived experience of mental illness. Considering research as an occupation can be important specifically for occupational scientists who study the concept of occupation itself and it can shed light on the ideas of occupation that are usually taken for granted. In Townsend's study (2000) focused on the clubhouse model, research was considered a meaningful occupation involving three opportunities for exploration: data collection and analysis, education, and action. Research as an important occupation was also discussed by Law (1997), who notes that providing opportunities for individuals to participate in the occupation of research may be considered as an important role for occupational therapists in community development (Law, 1997).

Our review also demonstrates that the experience of being a co-researcher may be challenging for persons living with mental illness.

Being unfamiliar with occupation of research (Bryant et al., 2010), fear and anxiety during the focus groups (Bryant et al., 2012), and technical difficulties are examples of challenges that persons living with mental illness experience as they engage in participatory studies (Maniam et al., 2016). It is important for occupational therapy and occupational science researchers and practitioners to consider the challenges that persons living with mental illness may face in the context of conducting participatory research and identify strategies for addressing some of these challenges to improve the experience for persons with lived experiences. Furthermore, if we want to engage persons living with mental illness in participatory research, we need to be willing to share power and control with persons with lived experiences as co-researchers.

Participatory research as a way of resisting stigma has the capacity to reveal the deeper psychological layers in the subjective experience of the stigma of mental illness and promote stigma resistance. The role of participatory research in promoting stigma resistance among persons with lived experiences of mental illness is documented in the literature (Rudnick, 2012; Whitley et al., 2020). Whitley et al. (2020) pointed out that participatory research is a feasible anti-stigma intervention and could be an effective means of stigma reduction. Rudnick (2012) also believed that stigma is a social injustice and argued that stigma could be decreased by involving persons with mental illness in the research process in which the expertise of these individuals is respected. There is a need within the field of occupational therapy and occupational science to consider the potential of participatory research to mitigate the traumatic impact of stigma among persons living with mental illness.

### Implications for Practice

Occupational therapy literature highlights that mental health consumers should be central to decision-making, and their choice should underpin their recovery process (Arblaster et al., 2018; Birken & Bryant, 2019). Participatory approaches align closely with recovery principles, which highlight the need for learning from lived experiences (Arblaster et al., 2019). Occupational therapy and occupational science could benefit from conducting research alongside persons with lived experiences in identifying occupational issues relevant to their lives, and in the co-design of novel interventions that are relevant and meaningful in the lives of persons living with mental illness.

Occupational therapists can find opportunities to collaborate with researchers and persons with lived experiences in conducts of participatory studies. We believe that investigating both the potentials and limitations of doing participatory research with mental health consumers will be insightful for researchers and it helps to achieve a more practical understanding of the participatory research approach.

### Implications for Research and Policy

Future occupational therapy and occupational science research in mental health should focus on conducting studies that involve persons with mental illness in the research and that provide refinements to practice. As the popularity of participatory methodologies grows, it is important to include critical reflection to find ways to overcome the obstacles and issues. It is worth mentioning that, in most of the included studies, participants were not engaged in all stages of the research. While it is challenging to incorporate all elements of participatory research into a single project (Gray et al., 2000), optimal outcomes can be achieved when occupational therapists and scientists embrace the values and principles of participatory research, ensuring a balanced power dynamic that maximizes the meaningful involvement of participants across all stages of the study.

There is also a need for policies at the university level to facilitate the process of conducting participatory research and avoid delays in obtaining ethical approval for projects where participants are also co-researchers. Funding opportunities are also needed for participatory projects to provide necessary resources to extend the use of participatory approaches in diverse settings and with diverse persons. We also need policies that support involving persons living with mental health in the design of new and re-design of the existing occupational therapy services to enhance the relevance of practice.

### Limitations

Several limitations in this study are acknowledged. First, we acknowledge that we could not distinguish between studies of varying degrees of quality by conducting a scoping review because scoping reviews do not involve a critical appraisal of included studies or the aggregation of data. Second, we reported the level of participation in the included studies based on the information provided by the authors. There is a possibility that some information related to the process of action and reflection in the included studies has been deleted due to word limitations. Third, although all the included studies were within the field of occupational therapy or occupational science, most of the authors did not discuss how participatory research can inform occupational therapy or occupational science. Fourth, the first author analyzed the data (EJ). She identifies as a Muslim woman without the lived experience of mental illness and recognizes that these social locations may have influenced the way that she analyzed and interpreted the findings of the included studies. Finally, most of the studies included in this review did not provide demographic characteristics of the participants such as clinical characteristics, disability, race, ethnicity, or the 2SLGBTQ + status. As a result, it would be difficult to comment on the ways in which the demographic characteristics of co-researchers in participatory projects impact the process of conducting research.

## Conclusion

The findings of this study underscore the importance of participatory research within the field of occupational therapy and occupational science with persons with lived and living experiences of mental illness. Involving individuals living with mental illness in all stages of research can help to shape the mental health system to become one that is more relevant, effective, and compassionate by incorporating the wisdom of persons with mental illness in its design and ongoing refinement. The findings of this study suggest that participatory approaches have been increasingly adopted by occupational therapy and occupational science to maximize the possibility that occupation-based services are relevant and meaningful for persons with mental illness. We hope that the findings of this review provide insights for researchers, practitioners, and persons with lived experiences about the use of occupation-focused participatory methods with persons living with mental illness and stimulate novel ideas for using these approaches with marginalized groups more commonly.

## Key Messages

- Participatory research by persons living with mental illness and occupational therapists can influence service delivery and design.
- Further investigation of the potential and challenges of occupation-focused participatory research could increase practical understanding.

## References

[bibr1-00084174231212760] Anderson Clarke L. M. Warner B. (2016). Exploring recovery perspectives in individuals diagnosed with mental illness. Occupational Therapy in Mental Health, 32(4), 400–418. 10.1080/0164212X.2016.1201450

[bibr2-00084174231212760] Andonian L. (2010). Community participation of people with mental health issues within an urban environment. Occupational Therapy in Mental Health, 26(4), 401–417. 10.1080/0164212X.2010.518435

[bibr3-00084174231212760] Andonian L. MacRae A. (2011). Well older adults within an urban context: Strategies to create and maintain social participation. British Journal of Occupational Therapy, 74(1), 2–11. 10.4276/030802211X12947686093486

[bibr4-00084174231212760] Arblaster K. Mackenzie L. Gill K. Willis K. Matthews L. (2019). Capabilities for recovery-oriented practice in mental health occupational therapy: A thematic analysis of lived experience perspectives. British Journal of Occupational Therapy, 82(11), 675–684. 10.1177/0308022619866129

[bibr5-00084174231212760] Arblaster K. Mackenzie L. Matthews L. Willis K. Gill K. Hanlon P. Laidler R. (2018). Learning from consumers: An eDelphi study of Australian mental health consumers’ priorities for recovery-oriented curricula. Australian Occupational Therapy Journal, 65(6), 586–597. 10.1111/1440-1630.12518 30221773

[bibr6-00084174231212760] Arksey H. O'Malley L. (2005). Scoping studies: Towards a methodological framework. International Journal of Social Research Methodology, 8(1), 19–32. 10.1080/1364557032000119616

[bibr7-00084174231212760] Ashby D. (1991). Practical statistics for medical research. Douglas G. Altman, Chapman and Hall, London, 1991. No. of pages: 611. Price: £ 32.00. In: Wiley Online Library.

[bibr8-00084174231212760] Baker A. E. Z. Procter N. G. (2014). Losses related to everyday occupations for adults affected by mental illness. Scandinavian Journal of Occupational Therapy, 21(4), 287–294. 10.3109/11038128.2014.894571 24666180

[bibr9-00084174231212760] Balcazar F. E. Keys C. B. Kaplan D. L. (2006). Participatory action research and people with disabilities: Principles and challenges.

[bibr10-00084174231212760] Balcazar F. E. Keys C. B. Kaplan D. L. Suarez-Balcazar Y. (1998). Participatory action research and people with disabilities: Principles and challenges. Canadian Journal of rehabilitation, 12(2), 105–112.

[bibr11-00084174231212760] Bassman R. (2001). Whose reality is it anyway? Consumers/survivors/ex-patients can speak for themselves. Journal of Humanistic Psychology, 41(4), 11–35. 10.1177/0022167801414002

[bibr12-00084174231212760] Berger S. (2011). The meaning of leisure for older adults living with vision loss. OTJR: Occupation, Participation and Health, 31(4), 193–199. 10.3928/15394492-20101222-01

[bibr13-00084174231212760] Bhagwanjee A. Stewart R. (1999). Promoting group empowerment and self-reliance through participatory research: A case study of people with physical disability. Disability and Rehabilitation, 21(7), 338–345. 10.1080/096382899297585 10471164

[bibr14-00084174231212760] Birken M. Bryant W. (2019). A Photovoice study of user experiences of an occupational therapy department within an acute inpatient mental health setting. British Journal of Occupational Therapy, 82(9), 532–543. 10.1177/0308022619836954

[bibr15-00084174231212760] Bryant W. Cordingley K. Adomako E. Birken M. (2019). Making activism a participatory, inclusive and developmental process: A research programme involving mental health service users. Disability & Society, 34(7–8), 1264–1288. 10.1080/09687599.2019.1613963

[bibr16-00084174231212760] Bryant W. Parsonage J. Tibbs A. Andrews C. Clark J. Franco L. (2012). Meeting in the mist: Key considerations in a collaborative research partnership with people with mental health issues. Work, 43(1), 23–31. 10.3233/WOR-2012-1444 22907320

[bibr17-00084174231212760] Bryant W. Vacher G. Beresford P. McKay E. (2010). The modernisation of mental health day services: Participatory action research exploring social networking. Mental Health Review Journal, 5(3), 11–21. 10.5042/mhrj.2010.0655

[bibr18-00084174231212760] Bryce H. (2012). Navigating multiple roles as a researcher in a Photovoice project. Groupwork, 22(3), 33–48. 10.1921/095182412X662176

[bibr19-00084174231212760] Cargo M. Mercer S. L. (2008). The value and challenges of participatory research: Strengthening its practice. Annual Review of Public Health, 29, 325–350. 10.1146/annurev.publhealth.29.091307.083824 18173388

[bibr20-00084174231212760] Chodo H. (2015). *Guidelines for Recovery-Oriented Practice. Hope. Dignity. Inclusion*. Retrieved from https://mentalhealthcommission.ca/wp-content/uploads/2021/05/MHCC_Recovery_Guidelines_2016_ENG.pdf

[bibr21-00084174231212760] Clark C. Scott E. Krupa T. (1993). Involving clients in programme evaluation and research: A new methodology for occupational therapy. Canadian Journal of Occupational Therapy, 60(4), 192–199. 10.1177/000841749306000405 10129021

[bibr22-00084174231212760] Cockburn L. Trentham B. (2002). Participatory action research: Integrating community occupational therapy practice and research. Canadian Journal of Occupational Therapy, 69(1), 20–30. 10.1177/000841740206900102 11852687

[bibr23-00084174231212760] Covidence systematic review software (2022). [Veritas Health Innovation]. www.covidence.org

[bibr24-00084174231212760] Dedoose (2021). (Version 9.0.17). www.dedoose.com

[bibr25-00084174231212760] Dixon K. Fossey E. Petrakis M. (2022). Using photovoice to explore women's experiences of a women-only prevention and recovery care service in Australia. Health & Social Care in the Community, 30(6), e5839–e5847. 10.1111/hsc.14015 PMC10087405 36069171

[bibr26-00084174231212760] Doll J. Brady K. (2013). Project HOPE: Implementing sensory experiences for suicide prevention in a Native American community. Occupational Therapy in Mental Health, 29(2), 149–158. 10.1080/0164212X.2013.788977

[bibr27-00084174231212760] Egan M. (2022). Promoting occupational participation: Collaborative relationship-focused occupational therapy (First ed.). CAOT/ACE.

[bibr28-00084174231212760] Egan M. Dubouloz C.-J. Rappolt S. Polatajko H. von Zweck C. King J. Vallerand J. Craik J. Davis J. A. Graham I. D. (2004). Enhancing research use through online action research. Canadian Journal of Occupational Therapy, 71(4), 230–237. 10.1177/000841740407100408 15586855

[bibr29-00084174231212760] Elliot J. (1991). Action research for educational change. McGraw-Hill Education (UK).

[bibr30-00084174231212760] Graneheim U. H. Lundman B. (2004). Qualitative content analysis in nursing research: Concepts, procedures and measures to achieve trustworthiness. Nurse Education Today, 24(2), 105–112. 10.1016/j.nedt.2003.10.001 14769454

[bibr31-00084174231212760] Gray R. E. Fitch M. Davis C. Phillips C. (2000). Challenges of participatory research: Reflections on a study with breast cancer self-help groups. Health Expectations, 3(4), 243–252. 10.1046/j.1369-6513.2000.00100.x 11281935 PMC5060116

[bibr32-00084174231212760] Greco V. Lambert H. C. Park M. (2017). Being visible: Photovoice as assessment for children in a school-based psychiatric setting. Scandinavian Journal of Occupational Therapy, 24(3), 222–232. 10.1080/11038128.2016.1234642 27665933

[bibr33-00084174231212760] Green L. W. Canada R. S. o. Research B. C. f. H. P. (1995). Study of participatory research in health promotion: Review and recommendations for the development of participatory research in health promotion in Canada.

[bibr34-00084174231212760] Ikiugu M. N. Schultz S. (2006). An argument for pragmatism as a foundational philosophy of occupational therapy. Canadian Journal of Occupational Therapy, 73(2), 86–97. 10.2182/cjot.05.0009 16680912

[bibr35-00084174231212760] Ingolia M. Barrett K. (2019). Mothers of children with complex trauma: Occupations viewed through photovoice. Occupational Therapy in Mental Health, 35(4), 339–360. 10.1080/0164212X.2019.1627977

[bibr36-00084174231212760] Jason L. A. Keys C. B. Suarez-Balcazar Y. E. Taylor R. R. Davis M. I. (2004). Participatory community research: Theories and methods in action. American Psychological Association.

[bibr37-00084174231212760] Kindon S. Pain R. Kesby M. (2007). Participatory action research approaches and methods. In Connecting people, participation and place (Vol. 260, p. 15). Routledge.

[bibr38-00084174231212760] Kleintjes S. R. (2012). Participation of people with psychosocial disability in mental health policy development in South Africa.

[bibr39-00084174231212760] Law M. (1997). Changing disabling environments through participatory action research: A Canadian experience. Nurtured by knowledge: Learning to do participatory action research, 34–58.

[bibr40-00084174231212760] Law M. Baptiste S. Mills J. (1995). Client-centred practice: What does it mean and does it make a difference? Canadian Journal of Occupational Therapy, 62(5), 250–257. 10.1177/000841749506200504 10152881

[bibr41-00084174231212760] Letts L. (2003). Occupational therapy and participatory research: A partnership worth pursuing. The American Journal of Occupational Therapy, 57(1), 77–87. 10.5014/ajot.57.1.77 12549893

[bibr42-00084174231212760] MacDonald C. (2012). Canadian journal of action research CJAR. Can J Action Res, 13(2), 34–50. 10.33524/cjar.v13i2.37

[bibr43-00084174231212760] Makdisi L. Blank A. Bryant W. Andrews C. Franco L. Parsonage J. (2013). Facilitators and barriers to living with psychosis: An exploratory collaborative study of the perspectives of mental health service users. British Journal of Occupational Therapy, 76(9), 418–426. 10.4276/030802213X13782044946346

[bibr44-00084174231212760] Manderscheid R. W. Ryff C. D. Freeman E. J. McKnight-Eily L. R. Dhingra S. Strine T. W. (2010). Peer reviewed: Evolving definitions of mental illness and wellness. Preventing Chronic Disease, 7(1), A19. PMCID: PMC2811514 PMC2811514 20040234

[bibr45-00084174231212760] Maniam Y. Kumaran P. Lee Y. P. Koh J. Subramaniam M. (2016). The journey of young people in an early psychosis program involved in participatory photography. British Journal of Occupational Therapy, 79(6), 368–375. 10.1177/0308022615621567

[bibr46-00084174231212760] McCabe R. Healey P. G. Priebe S. Lavelle M. Dodwell D. Laugharne R. Snell A. Bremner S. (2013). Shared understanding in psychiatrist–patient communication: Association with treatment adherence in schizophrenia. Patient Education and Counseling, 93(1), 73–79. 10.1016/j.pec.2013.05.015 23856552

[bibr47-00084174231212760] Mirza M. Gossett A. Chan N. K. C. Burford L. Hammel J. (2008). Community reintegration for people with psychiatric disabilities: Challenging systemic barriers to service provision and public policy through participatory action research. Disability & Society, 23(4), 323–336. 10.1080/09687590802038829

[bibr48-00084174231212760] O’Brien J. Fossey E. Palmer V. J. (2021). A scoping review of the use of co-design methods with culturally and linguistically diverse communities to improve or adapt mental health services. Health & Social Care in the Community, 29(1), 1–17. 10.1111/hsc.13105 32686881

[bibr49-00084174231212760] Papageorgiou A. Loke Y. K. Fromage M. (2017). Communication skills training for mental health professionals working with people with severe mental illness. Cochrane Database of Systematic Reviews, (6). 10.1002/14651858.CD010006.pub2 PMC6481374 28613384

[bibr50-00084174231212760] Pettican A. Speed E. Bryant W. Kilbride C. Beresford P. (2022). Levelling the playing field: Exploring inequalities and exclusions with a community-based football league for people with experience of mental distress. Australian Occupational Therapy Journal, 69(3), 290–300. 10.1111/1440-1630.12791 35067953 PMC9304312

[bibr51-00084174231212760] Pettican A. Speed E. Kilbride C. Bryant W. Beresford P. (2021). An occupational justice perspective on playing football and living with mental distress. Journal of Occupational Science, 28(1), 159–172. 10.1080/14427591.2020.1816208

[bibr52-00084174231212760] Rempfer M. Knott J. (2002). Participatory action research: A model for establishing partnerships between mental health researchers and persons with psychiatric disabilities. Occupational Therapy in Mental Health, 17(3-4), 151–165. 10.1300/J004v17n03_10

[bibr53-00084174231212760] Rudnick A. (2012). Recovery of people with mental illness: Philosophical and related perspectives. Oxford University Press.

[bibr54-00084174231212760] Russinova Z. Mizock L. Bloch P. (2018). Photovoice as a tool to understand the experience of stigma among individuals with serious mental illnesses. Stigma and Health, 3(3), 171. 10.1037/sah0000080

[bibr55-00084174231212760] Rustage K. Crawshaw A. Majeed-Hajaj S. Deal A. Nellums L. Ciftci Y. Fuller S. S. Goldsmith L. Friedland J. S. Hargreaves S. (2021). Participatory approaches in the development of health interventions for migrants: A systematic review. BMJ Open, 11(10), e053678. 10.1136/bmjopen-2021-053678 PMC8548676 34697122

[bibr56-00084174231212760] Schwartz A. E. Team Y. A. M. H. P. M. R. Kramer J. M. Rogers E. S. McDonald K. E. Cohn E. S. (2020). Stakeholder-driven approach to developing a peer-mentoring intervention for young adults with intellectual/developmental disabilities and co-occurring mental health conditions. Journal of Applied Research in Intellectual Disabilities, 33(5), 992–1004. 10.1111/jar.12721 32119173 PMC11611146

[bibr57-00084174231212760] Schwartz R. Estein O. Komaroff J. Lamb J. Myers M. Stewart J. Vacaflor L. Park M. (2013). Mental health consumers and providers dialogue in an institutional setting: A participatory approach to promoting recovery-oriented care. Psychiatric Rehabilitation Journal, 36(2), 113. 10.1037/h0094980 23750763

[bibr58-00084174231212760] Shaffer E. J. Lape J. E. Salls J. (2020). Decreasing stress for parents of special needs children through a web-based mindfulness program: A pilot study. Internet Journal of Allied Health Sciences and Practice, 18(4), 16. 10.46743/1540-580x/2020.1887

[bibr59-00084174231212760] Smith S. Suto M. J. (2014). Spirituality in bedlam: Exploring patient conversations on acute psychiatric units: La spiritualité dans le contexte des unités de soins intensifs psychiatriques: Explorer les conversations des patients sur la spiritualité. Canadian Journal of Occupational Therapy, 81(1), 8–17. 10.1177/0008417413516932 24783484

[bibr60-00084174231212760] Stacciarini J.-M. R. Shattell M. M. Coady M. Wiens B. (2011). Review: Community-based participatory research approach to address mental health in minority populations. Community Mental Health Journal, 47(5), 489–497. 10.1007/s10597-010-9319-z 20464489

[bibr61-00084174231212760] Suarez-Balcazar Y. Early A. Maldonado A. Garcia C. P. Arias D. Zeidman A. Agudelo-Orozco A. (2018). Community-based participatory research to promote healthy lifestyles among Latino immigrant families with youth with disabilities. Scandinavian Journal of Occupational Therapy, 25(5), 396–406. 10.1080/11038128.2018.1502348 30280951

[bibr62-00084174231212760] Suto M. J. Smith S. (2014). Spirituality in bedlam: Exploring professional conversations on acute psychiatric units: La spiritualité dans le contexte des unités de soins intensifs psychiatriques: Explorer les conversations des professionnels sur la spiritualité. Canadian Journal of Occupational Therapy, 81(1), 18–28. 10.1177/0008417413516931 24783485

[bibr63-00084174231212760] Suto M. J. Smith S. Damiano N. Channe S. (2021). Participation in community gardening: Sowing the seeds of well-being: Participation au jardinage communautaire: Pour semer les graines du bien-être. Canadian Journal of Occupational Therapy, 88(2), 142–152. 10.1177/0008417421994385 PMC8240003 33761777

[bibr64-00084174231212760] Townsend E. Birch D. E. Langley J. Langille L. (2000). Participatory research in a mental health clubhouse. The Occupational Therapy Journal of Research, 20(1), 18–44. 10.1177/153944920002000102

[bibr65-00084174231212760] Tricco A. C. Lillie E. Zarin W. O'Brien K. K. Colquhoun H. Levac D. Moher D. Peters M. D. Horsley T. Weeks L. (2018). PRISMA extension for scoping reviews (PRISMA-ScR): Checklist and explanation. Annals of Internal Medicine, 169(7), 467–473. 10.7326/M18-0850 30178033

[bibr66-00084174231212760] Tsatsi I. A. Plastow N. A. (2021). Optimizing a Halfway House to Meet Mental Health Care Users’ Occupational Needs. Canadian Journal of Occupational Therapy, 88(4), 352–364. 10.1177/00084174211044896 34709087

[bibr67-00084174231212760] Wang C. C. (1999). Photovoice: A participatory action research strategy applied to women's health. Journal of Women's Health, 8(2), 185–192. 10.1089/jwh.1999.8.185 10100132

[bibr68-00084174231212760] Whitley R. Sitter K. C. Adamson G. Carmichael V. (2020). Can participatory video reduce mental illness stigma? Results from a Canadian action-research study of feasibility and impact. BMC psychiatry, 20(1), 1–12. 10.1186/s12888-020-2429-4 31918689 PMC6953159

[bibr69-00084174231212760] Whyte W. F. (1998). Rethinking sociology: Applied and basic research. The American Sociologist, 16–19. 10.1007/s12108-998-1016-9

[bibr169-00084174231212760] Wimpenny K. Forsyth K. Jones C. Matheson L. Colley J. (2010). Implementing the Model of Human Occupation across a mental health occupational therapy service: Communities of practice and a participatory change process. British Journal of Occupational Therapy, 73(11), 507–516. 10.4276/030802210X12892992239152

